# Obstacles and approaches in artificial cultivation of Chinese cordyceps

**DOI:** 10.1080/21501203.2018.1442132

**Published:** 2018-03-05

**Authors:** Qi-lian Qin, Gui-ling Zhou, Huan Zhang, Qian Meng, Ji-hong Zhang, Hong-tuo Wang, Lin Miao, Xuan Li

**Affiliations:** State Key Laboratory of Integrated Management of Pest Insects and Rodents, Institute of Zoology, Chinese of Academy of Sciences, Beijing, China

Chinese cordyceps, also known as “Dong Chong Xia Cao” in Chinese, which means being worm in winter and herb in summer, is the fruiting body of *Ophiocordyceps sinensis* developed from the cadaver mummy (sclerotium) of infected ghost moth larva distributed in the Qinghai–Tibetan Plateau around the altitude of 3000–5000 m (Zhang et al. 2012). It is a well-known traditional Chinese medicine mostly because of its legendary miraculous beneficial effects to human health and its very high prices in the market, which reached as high as USD 60,000 per kg in 2015 (Lei et al. 2015). Almost all the Chinese cordyceps in the market are harvested in the alpine pasture of the Qinghai–Tibetan Plateau during the period of May to July every year, which severely damage the pasture vegetation and ecology of the plateau. A lot of institutions and companies have been investing huge resources to explore artificial cultivation of Chinese cordyceps indoor or in its natural habitat for a long time, but failed until recent because of difficulties of artificially completing both life cycles of host insect and fungal infection in the host involved in the cultivation. It is known that several institutions and companies have been successful in artificial cultivation of Chinese cordyceps. However, there is no formal scientific report on the details of this success, which is probably because of huge commercial interests behind the area and technical know-hows in which developers do not want to share with others. In this short note, we briefly present some technical bottlenecks retarding the progress of artificial cultivation of Chinese cordyceps empirically and list some examples of the success from personal communications. It is a need to illustrate that the information provided below is more empirical than scientific.

## 1. Long life cycle makes it difficult to rear the host insect artificially indoors on large scale

The hosts of *O. sinensis* are larvae of the ghost moths (Hepialidae, Lepidoptera) that exclusively live in the soil under pasture of the Tibetan Plateau with a life cycle of 3 to 5 years. This long time could be shortened to ca. 1 year in favourable controlled conditions. However, the shortened life cycle is still too long for large-scale rearing, considering numerous pathogens of the insect threatening the population except *O. sinensis*. Also, the cryptic habitat of the insect makes it difficult to uncover its biology. It is essential to maintain both high quality and quantity of the host population for further artificial cultivation of Chinese cordyceps.

## 2. Highly infectious pathogens of the host could lead to disastrous impact to the artificial cultivation

**Figure 1.**
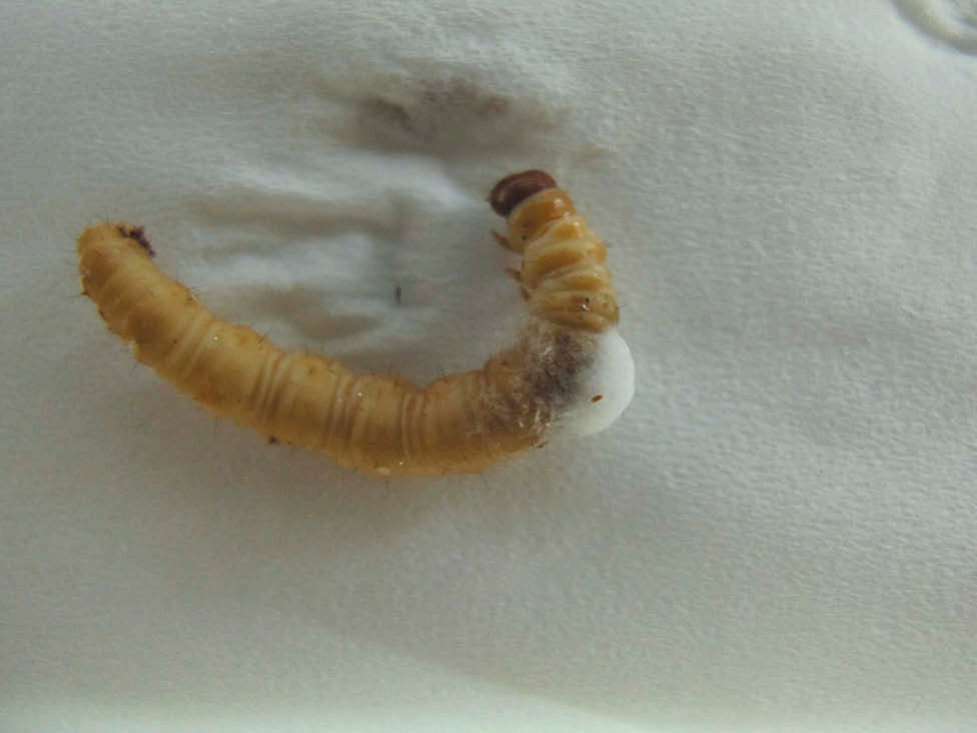
A *Thitarodes xiaojinensis* larva killed by *Paecilomyces farinosus.*

There are a lot of lethal pathogens which could infect and kill the host insect during the cultivation. In our experiences and experiments, the most fatal pathogen is *Paecilomyces* sp. We have used ITS (Internal Transcribed Spacer) DNA marker to analyse isolates from 148 ghost moth (*Thitarodes xiaojinensis*) cadavers killed by microbes during the cultivation and found that 122 samples were killed by *Paecilomyces farinosus* (Table 1 and Figure 1, unpublished data), a famous entomopathogenic fungus used in pest insect biological control. This result corresponds with other personal communications. Eradication of contamination of *P. farinosus* and other pathogens among the host population is an arduous task for the cultivation.

**Table 1.** ITS analysis of isolates from infected *Thitarodes xiaojinensis* cadavers.

Fungal species	Number (totally 148)	Proportion (%)
*Paecilomyces farinosus*	122	85.92
*Beauveria bassiana*	3	2.11
*Penicillium polonicun*	8	5.63
*Penicillium commune*	4	2.82
*Penicillium expansum*	4	2.82
*Tritirachium* sp.	3	2.11
*Galactomyces geotrichum*	1	0.70
*Fusarium* sp.	1	0.70
*Mucor circinelloides*	1	0.70
*Mucor hiemalis*	1	0.70

## 3. Mechanism of how *O. sinensis* invading the host is obscure

*O. sinensis* is an entomopathogenic fungus that must penetrate into and finally kill its host to complete its life cycle. How, when and by what the host is infected is not understood very well. From available literatures, we can find that conidia, mycelia and ascospores of the fungus were believed as the entities to infect the insects, respectively. Treatment of spray, feeding orally and/or puncture with the respective inocula to different host stadia were adopted by various authors. However, efficiency of inoculation was usually unsatisfied. Although we are working on the host immunology against *O. sinensis* (cover picture), there are few reports concerning physiological and immunological interactions between the fungus and its host, which are very important for understanding the mechanism of infection and development of the fungus in its host and essential for large-scale cultivation at low cost.

## 4. Huge economic interests in the cultivation hindered technical research and development of its industrialisation and commercialisation

A lot of problems in research and development are more economic than scientific. High price and miraculous functions for human health of Chinese cordyceps imply huge economic interests behind its large-scale cultivation. Almost all developers do the work on their own independently and are not willing to share their progress and technical know-hows with others, which restrains the exchange of achievements among developers and severely impairs development of its industrialisation and commercialisation as a whole.

## 5. Some successful examples of the artificial cultivation

**Figure 2.**
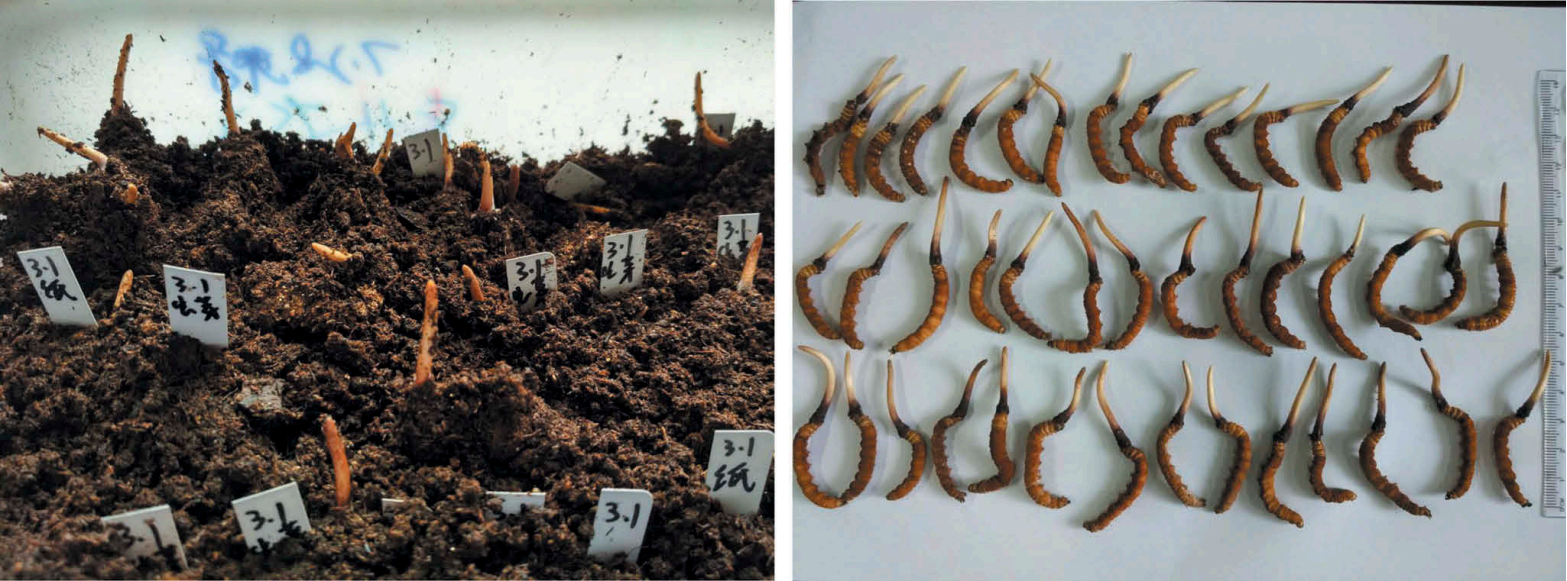
Fruiting bodies of *Ophiocordyceps sinensis* growing out from base material under artificial conditions (left); Chinese cordyceps cultivated in Institute of Zoology, Chinese Academy of Sciences (right).

Although there are a lot of difficulties across the road to cultivate Chinese cordyceps artificially, many progresses have been achieved in the area, including success in artificial cultivation on large scale (Dong 2016). Yichang Shanchengshuidu Cordyceps Co., LTD had established a developing and producing base of Chinese cordyceps in Yidu county, Hubei province (Li et al. 2016), and reached an annual yield of 15 tons in 2017 (personal communication). Other institutions had reproducibly completed the life cycle of *O. sinensis* from anamorph to teleomorph in its host, including groups from Guangdong Institute of Applied Biological Resources led by Prof. Richou Han (Qiu et al. 2016), Xishuangbanna Tropical Botanical Garden, Chinese Academy of Sciences led by Prof. Darong Yang and Sichuan Agricultural University led by Prof. Zuji Zhou (personal communications). Our group also had got the success in 2016 (Figure 2). These progresses must enormously promote industrialisation and commercialisation of artificial cultivation of Chinese cordyceps. It is said that some institutions and companies also attempt to cultivate the fruiting bodies of *O. sinensis* on artificial substrates other than host insects and make some progress.

## 6. Challenges and perspectives

The mysterious aureole shrouds on Chinese cordyceps has been revealed along with the success of its artificial cultivation after several decades of developing history. To date, more and more institutions and companies begin to engage in the domain. However, there are a lot of challenges set before developers and producers: (1) How to reduce the rearing cost of the ghost moth, the fungal host, on large scale is crucial for the industrialisation; (2) How to inhibit disastrous infections from other entomopathogenic microbes during long periods (usually more than 1 year) of the cultivation is not well resolved. (3) Mechanisms of physiological and immunological interactions between the fungus and its host, including how the fungus infects and penetrates into the host, what immune response the host imposes onto the fungus and how the fungus develops within and grows out from the infected host body (sclerotium), should be elucidated. (4) Impact of the increasing productivity of artificial cultivated Chinese cordyceps on the market should be cautiously considered.

The tonic and medical functions of Chinese cordyceps, as well as cultivated fruiting bodies on artificial substrates and fermented mycelia for human body, determine a huge market demand in the world, especially regions influenced by the culture of traditional Chinese medicine. Annual output of excavated Chinese cordyceps in the plateau is far away from the market needs. From points of view of protecting ecology and environment of the Qinghai–Tibetan Plateau and matching the market needs, mass production of artificially cultivated Chinese cordyceps is an urgent and a very important task.
